# Evaluation of Surgi‐ORC for Hemostasis and Safety in Gynecological Surgeries: A Multicenter Prospective Observational Pilot Study

**DOI:** 10.1155/ogi/5056815

**Published:** 2026-09-01

**Authors:** Diksha Sharma, Bhavin Trivedi, Shahid N. Karatela, Piyush Patel

**Affiliations:** ^1^ Department of Research and Development, Aegis Lifesciences Pvt. Ltd., Ahmedabad Gujarat, 382213, India; ^2^ Department of Regulatory Affairs, Aegis Lifesciences Pvt. Ltd., Ahmedabad Gujarat, 382213, India; ^3^ Department of Quality, Aegis Lifesciences Pvt. Ltd., Ahmedabad Gujarat, 382213, India

**Keywords:** gynecological surgery, hemostasis, observational study, oxidized regenerated cellulose, Surgi-ORC, surgical bleeding control, topical hemostatic agent

## Abstract

**Background and Aims:**

Achieving adequate intraoperative hemostasis is critical in gynecological surgeries to reduce perioperative morbidity. Surgi‐oxidized regenerated cellulose (ORC) is a biodegradable hemostatic agent that controls mild to moderate bleeding. This study aimed to generate preliminary observational data on the efficacy and safety of Surgi‐ORC across a range of gynecological procedures.

**Methods:**

This prospective, multicentre, single‐arm observational study was conducted at two centres in India. A total of 28 women undergoing gynecological surgeries, including hysterectomy, myomectomy, sacrohysteropexy, cystectomy with oophorectomy, laparotomy, and recanalization, received Surgi‐ORC intraoperatively for bleeding control. Time to hemostasis (TTH), the association between product variants and TTH, the association of TTH across surgery types, short‐term safety outcomes, and surgeon‐reported usability were assessed. Follow‐ups were conducted on postoperative Days 2, 28, and 60. Subgroup analyses were considered exploratory due to the limited sample size.

**Results:**

Hemostasis was achieved in all cases, with a mean TTH of 1.3 min (SD = 0.43). Descriptive evaluation showed broadly similar TTH values across surgery types and product variants; however, small and imbalanced subgroup sizes limited definitive conclusions. On Day 2, X‐ray imaging showed no radiographic abnormalities attributable to retained material and was confirmed based on clinical judgment of the radiologist and operating surgeon. No device‐related adverse events, complications, or short‐term safety concerns were identified during the study period. Most surgeons rated handling characteristics as “Good” across all evaluated parameters, indicating favorable usability.

**Conclusion:**

This observational pilot study suggests that Surgi‐ORC is effective in achieving hemostasis and demonstrates no short‐term safety concerns during the 60‐day follow‐up along with satisfactory usability characteristics in gynecological procedures. However, given the exploratory nature of analysis, single‐arm design, and small sample size, these findings should be interpreted cautiously, and larger comparative studies are required to further validate its clinical utility.

## 1. Introduction

Gynecological surgeries including hysterectomy, myomectomy, and ovarian cystectomy involve manipulating significant vascular structures and tissues, which can result in potential intraoperative blood loss, prolonged operative time, increased blood transfusion requirement, and postoperative morbidity [1, 2]. Hence, proper intraoperative hemostasis is necessary to enhance the outcome of the surgery and reduce the number of postoperative complications—such as hematoma formation and infections.

Many conventional hemostatic methods such as electrocautery, suturing, and mechanical compression are commonly used but likely to be inadequate in diffuse or oozing bleeding in highly vascularized tissues [3, 4]. To address these challenges, adjunctive hemostatic agents, such as fibrin sealants, thrombin‐based products, and oxidized regenerated cellulose (ORC)‐based materials, have been introduced. Among them, hemostatic agents based on ORC have received a growing interest due to their biodegradability and capability to promote clot formation by local activation of the coagulation cascade. [5, 6]. In addition, it has been reported that the use of ORC across different surgical contexts including gynaecological procedures have generally demonstrated positive results in terms of patient satisfaction and functional outcome [7–10].

ORC is a plant‐based hemostatic agent that regenerates pure cellulose into an interwoven fabric, followed by oxidation. Its biodegradable nature allows it to be left in situ, making it convenient for intraoperative use. It also offers antimicrobial activity, addressing a promising surgical site infection (SSI) management alternative [11]. It is also associated with a lower risk of adhesions, making it particularly suitable for gynecological procedures where delicate tissue handling is essential [12].

Surgi‐ORC, an ORC‐based hemostatic agent, has been developed for localized control of mild to moderate bleeding and is available in different variants to accommodate surgical needs. Despite its growing use in various surgical disciplines, preliminary evidence on the clinical effectiveness and safety of Surgi‐ORC in gynecological procedures has not been extensively evaluated. Recent studies have primarily focused on its use in orthopedic surgeries [13], leaving a gap in the literature regarding its specific benefits in gynecological surgeries.

To bridge this gap, this prospective, multicentre, observational pilot study was conducted to generate preliminary data on the effectiveness, safety, and usability characteristics of Surgi‐ORC in gynecological surgeries.

## 2. Methodology

### 2.1. Study Design and Settings

This prospective, multicentre, single‐arm, observational pilot study without a control arm was conducted to gather preliminary data on the clinical performance and safety of Surgi‐ORC in routine gynecological surgeries. The study was conducted at two institutions in India: (1) Jasleen Hospital, Nagpur, and (2) Shri Shankaracharya Institute of Medical Sciences, Bhilai. The study was registered with the Clinical Trials Registry of India (CTRI/2024/02/062445‐ORTH/002), ensuring transparency and adherence to national research standards. All procedures were conducted in compliance with ICH‐Good Clinical Practice, the Declaration of Helsinki, ISO 14155, and MDR (EU) 2017/745. The study was conducted in accordance with STROBE principles for nonrandomized observational studies.

### 2.2. Participants/Patient Selection Criteria

Patients eligible for inclusion in this study were women aged ≥ 18 years undergoing elective or emergency gynecological surgeries in which intraoperative hemostasis was deemed necessary at the discretion of the operating surgeon. These procedures included, but were not limited to, hysterectomy, myomectomy, ovarian cystectomy, sacrohysteropexy, oophorectomy, and other laparoscopic or open pelvic surgeries. This study included patients willing to participate, able to provide informed consent, and scheduled for a gynecological procedure in which Surgi‐ORC could be used for management of intraoperative hemostasis. Patients with known hypersensitivity to ORC or any components of Surgi‐ORC were excluded. Additional exclusion criteria included severe systemic infections, uncontrolled coagulopathies, active infection at the surgical site, and those diagnosed with known bleeding disorders (e.g., thrombocytopenia, thrombasthenia, hemophilia, or von Willebrand disease) or those enrolled in another interventional clinical trial that could interfere with the outcomes of this study. Pregnant or lactating women were also excluded unless there was no contradiction for surgery and written informed consent was obtained.

### 2.3. Sample Size Determination

Given the exploratory nature of the study, without a control arm, no formal sample size calculation was performed. Instead, the number of participants was determined pragmatically based on the expected patient flow at the participating centres and the feasibility of enrolment within the defined study period. A total of 28 participants were enrolled during the study period. The findings should be interpreted accordingly to the sample size of the study.

### 2.4. Participants/Surgical Procedure

Participants undergoing gynecological procedures were enrolled consecutively at the two participating centres. Surgi‐ORC was applied intraoperatively at the site(s) of active bleeding, as determined by the operating surgeon as per standard surgical practice. The product was applied directly to the bleeding surface and left in place as per the manufacturer’s instructions provided with the product. Details regarding the type of surgery, sites of application, degree of bleeding, wound size, blood volume, and model and number of Surgi‐ORC used were documented for each patient.

### 2.5. Outcomes/Endpoints

The primary outcome of the study was the efficacy of Surgi‐ORC in achieving intraoperative hemostasis. Time to hemostasis (TTH), defined as the time required to achieve successful control of bleeding, was assessed and recorded by the operating surgeon based on visual observation and clinical thinking up to a maximum of 10 min. The ≤ 5‐min threshold was selected as the predefined criteria for successful hemostasis based on clinically acceptable timeframes for ORC‐based topical hemostatic agents reported in literature. Additionally, hemostasis achieved under 2 min was reported descriptively to highlight rapid bleeding control rather than using it as a separate predefined efficacy endpoint. Secondary outcomes included postoperative short‐term safety endpoints such as the incidence of clinically relevant complications, including SSIs, hematoma, seroma formation, and delayed bleeding through a 60‐day follow‐up period. Postoperative X‐ray imaging was performed as part of routine clinical assessment; however, confirmation of absorption using validated imaging modalities was not performed.

In addition, surgeon‐reported outcomes related to ease of application, ease of delivery to the wound site, ease of preparation, conformance to the tissue surface, and overall handling characteristics of the product were collected using a nonvalidated, study‐specific questionnaire and are considered subjective.

### 2.6. Data Collection and Follow‐Up

Data were recorded by operating surgeons and study coordinators using a structured case record form designed for this study. They documented comprehensive clinical information, including patient demographics, type of gynecological procedure performed, bleeding characteristics, the variant of Surgi‐ORC used, site and dimension of application site (wound), TTH, and any immediate intraoperative observations. Postoperative data were also collected, focusing on recovery progress, follow‐up, adverse events, and overall surgical outcomes. At clinical sites, participants were monitored throughout the 28‐day postoperative period for any adverse events related to the use of Surgi‐ORC. Follow‐up assessments were limited to 60 days postsurgery to monitor short‐term safety outcomes of the product. Laboratory investigations were conducted during follow‐up visits when site investigators deemed necessary.

### 2.7. Bias and Study Limitations

The study has several methodological limitations. First, all authors are employees of the manufacturer of Surgi‐ORC, and no independent clinician investigators from participating centres were included as co‐authors. Although clinical procedures, patient management, and clinical outcome assessments were performed independently by site investigators according to routine clinical practice, the possibility of sponsor‐related reporting or interpretation bias cannot be completely excluded. Second, surgeon‐reported usability outcomes were subjective in nature and were not independently adjudicated or assessed using validated instruments. Third, the single arm design without a control group and the small and uneven subgroup sizes, along with short‐term 60‐day follow‐up period, may limit the interpretation and generalizability of the findings. Future studies should incorporate larger patients, independent investigators, a comparator group, and validated usability measures to further enhance objectivity and generalizability.

### 2.8. Ethical Approval and Informed Consent

This study was conducted in accordance with applicable national laws, the Declaration of Helsinki, and Good Clinical Practice guidelines. Ethical approval was obtained prior to study initiation from the Jasleen Hospital Ethics Committee, Jasleen Hospital, Nagpur, Maharashtra, India, and the Institutional Ethics Committee of Shri Shankaracharya Institute of Medical Sciences, Junwani, Bhilai, Durg, Chhattisgarh, India. Written informed consent was obtained from all participants or their legal guardians wherever applicable, after providing clear information about the study’s purpose, procedures, potential risks, and benefits. Participants were free to withdraw at any time without consequences. Confidentiality and anonymity of participant data were strictly maintained throughout the study.

### 2.9. Statistical Analysis

Descriptive statistics were used to summarize patient demographics, types of surgeries, and outcome measures. Categorical variables were expressed as frequencies and percentages, while continuous variables are summarized using mean ± standard deviation (SD) or median (interquartile range [IQR]), as appropriate.

The primary outcome, TTH, was descriptively summarized for each surgical procedure and product variant. A chi‐square test was performed to assess the association between the type of surgery and bleeding severity. Because this was a pilot observational study, no formal inferential subgroup analysis was performed due to the exploratory nature of the study and the small, unequal sizes of subgroups. Data were analyzed using IBM SPSS Statistics for Windows, Version 25.0 (IBM Corp., Armonk, NY, USA). The reporting of statistical results was aligned with SAMPL guidelines to ensure transparency and completeness [14].

## 3. Results

### 3.1. Demographics and Gynecological Procedure

A total of 28 patients were enrolled in the study, with a mean (SD) age of 41 years (9.29). The most common gynecological procedure performed was hysterectomy (*n* = 17), followed by cystectomy and oophorectomy (*n* = 4), myomectomy (*n* = 2), sacrohysteropexy (*n* = 2), recanalization (*n* = 2), and laparotomy (*n* = 1). A summary of patient demographics and gynecological surgery procedures is presented in Table 1.

**TABLE 1:** Demographic and patient characteristics.

Parameters	Patients, *N* = 28
Age, years	
Mean (SD)	40.75 (9.29)
Median (IQR)	43.5 (34.5–46)
Gynaecological Procedures	
Hysterectomy, *n* (%)	17 (60.71%)
Cystectomy and oophorectomy, *n* (%)	4 (14.28%)
Myomectomy, *n* (%)	2 (7.14%)
Sacrohysteropexy, *n* (%)	2 (7.14%)
Recanalization, *n* (%)	2 (7.14%)
Laparotomy, *n* (%)	1 (3.57%)

### 3.2. Intraoperative Bleeding Characteristics and Surgi‐ORC Variant Usage

Intraoperative bleeding characteristics varied among patients, with moderate bleeding observed in 75% (*n* = 21) and mild bleeding in 25% (*n* = 7) of the cases. The average dimension of the bleeding site was 7.63 cm, with a median of 6.75 cm (range: 3.57–10). The mean (SD) estimated intraoperative blood loss was 46.2 mL (21.07), with a range of 1.5 mL–75 mL. The median blood loss was 55 mL (IQR: 17.5 mL). Notably, no patient experienced blood loss exceeding 100 mL, and no intraoperative transfusions were required.

Based on clinical judgement by surgeons, different product variants were used during the surgical procedures, and among them, in two instances, more than one product was applied to manage bleeding. The primary endpoint was to evaluate the TTH and the proportion of wounds achieving hemostasis at 2, 5, and 10 min after the application of product. All patients (100%) achieved hemostasis within 5 min, and 85.71% (24 of 28) achieved hemostasis in less than 2 min. Hemostasis achieved under 2 min is reported descriptively to represent observation of rapid bleeding control of Surgi‐ORC. These results remained within the predefined success criteria of ≤ 5 min but should not be interpreted as a separate efficacy endpoint. The descriptive TTH values across different product variants are detailed in Table 2. The overall mean TTH recorded across all cases was 1.3 min (SD = 0.43).

**TABLE 2:** Descriptive summary of TTH across different product variants.

Device variant	Mean (SD) TTH in minutes^a^
Surgi‐ORC Original/Standard (*n* = 11)	0.85 (0.24)
Surgi‐ORC Nonwoven/Snow (*n* = 7)	0.44 (0.43)
Surgi‐ORC Fibril (*n* = 1)	NA^b^
Surgi‐ORC Knit (*n* = 9)	0.86 (0.33)

^a^Comparisons across device variants are descriptive only.

^b^Only one patient had the application with Surgi‐ORC Fibril.

### 3.3. Association Between Type of Surgery and Bleeding Characteristics

The distribution of bleeding severity (mild vs. moderate) across various gynecological procedures is summarized in Table 3. The most frequent procedure performed was hysterectomy, where 14 out of 17 patients experienced moderate bleeding. In contrast, procedures such as laparotomy and myomectomy showed a more balanced distribution between mild and moderate bleeding. A chi‐square test yielded a chi‐square statistic of 6.82 with 5 degrees of freedom and a *p* value of 0.234. This indicates that there is no statistically significant association between the type of surgical procedure and the degree of bleeding (*p* > 0.05).

**TABLE 3:** Distribution of surgery types and intraoperative bleeding characteristics.

Surgery procedure	Mild bleeding (*n*)	Moderate bleeding (*n*)
Cystectomy and Oophorectomy	2	2
Hysterectomy	3	14
Myomectomy	1	1
Exploratory Laparotomy	1	0
Recanalization	0	2
Sacrohysteropexy	0	2

### 3.4. Efficacy

#### 3.4.1. TTH Across Surgi‐ORC Variants

TTH was descriptively evaluated among the four product variants, including Nonwoven/SNOW, Original/Standard, Fibril, and Knit. The Fibril variant (*n* = 1) was not included in comparative analysis due to insufficient sample size. Median and mean TTH values revealed some variability across variants; however, given the small and uneven subgroup analysis for product variants, no formal statistical comparisons were considered reliable. So, variant‐wise differences in TTH should be interpreted as exploratory and hypothesis‐generating only. No definitive conclusions can be made for efficacy between product variants based on the present dataset.

#### 3.4.2. Association of TTH Across Different Surgical Procedures

**FIGURE 1:**
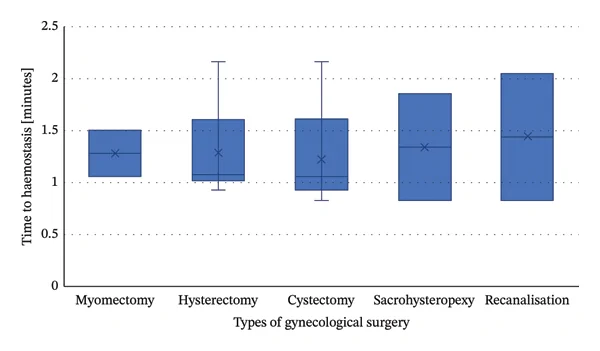
Boxplot of TTH by types of gynecological surgery.

TTH was descriptively evaluated across different gynecological procedures. However, several procedure categories included only one or two patients, limiting meaningful comparisons. Boxplot as illustrated in Figure 1, represents TTH distributions across surgical procedures, with overlapping IQR. Hysterectomy cases, which constituted the majority of the cohort, showed a consistent distribution of TTH with moderate variability. Descriptively, TTH values appeared to vary across surgical procedures. However, given the small and uneven subgroup sizes, particularly for procedures with ≤ 2 cases, these findings are descriptive and exploratory in nature and do not indicate definitive procedure‐specific differences.

### 3.5. Safety

**FIGURE 2:**
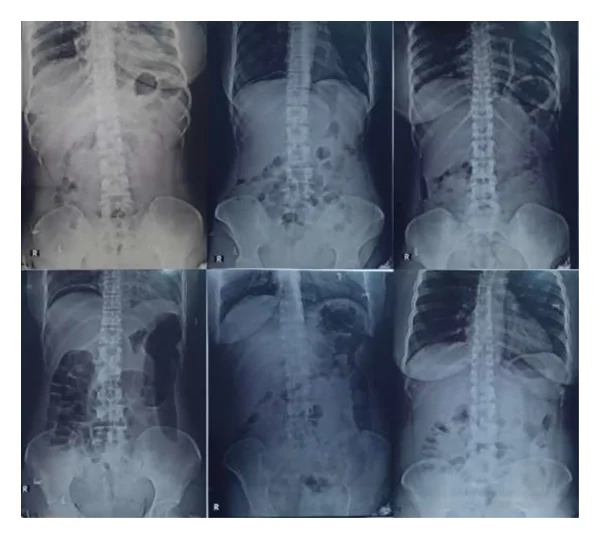
Radiological imaging on Day 2 postimplantation of Surgi‐ORC.

The safety assessment was based on postoperative outcomes and the presence of any clinically evident complications following implantation of product during gynecological surgeries. All 28 patients underwent radiological imaging on postoperative Day 2 as part of routine clinical assessment, and no radiographic abnormalities attributable to retained material were observed (Figure 2). This conclusion was made based on the absence of imaging findings suggestive of retained material, together with the clinical assessments of the radiologist and operating surgeon. It is important to note that plain radiography is not a validated modality for detecting ORC.

Throughout the postoperative observation period, no clinically evident SSIs or device‐related foreign body reactions were reported. All patients demonstrated satisfactory postoperative recovery and wound healing, with no short‐term safety concerns related to the device. These findings are limited to short‐term postoperative outcomes of Surgi‐ORC in gynecological surgical settings within the follow‐up period evaluated. Long‐term safety outcomes such as adhesion formation or delayed foreign‐body reactions beyond 60 days were not evaluated in this study.

### 3.6. Handling Characteristics of Surgi‐ORC

**FIGURE 3:**
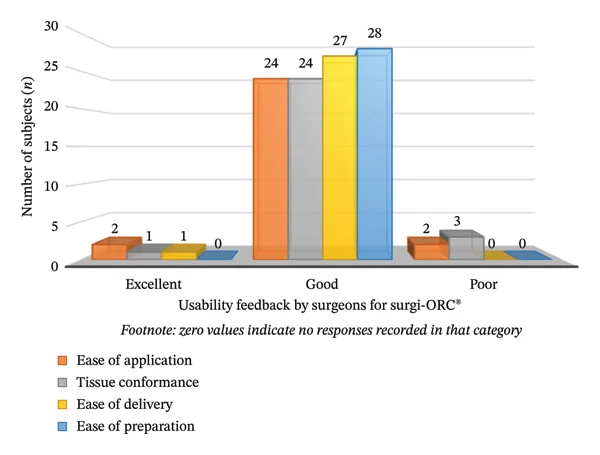
Evaluation of intraoperative handling characteristics of Surgi‐ORC based on surgeons’ feedback (*n* = 28).

The usability and intraoperative handling characteristics of Surgi‐ORC were evaluated across four predefined domains: ease of application to the bleeding site, conformance to the tissue surface, ease of delivery to the wound site, and ease of handling for use. The overall assessment demonstrated favorable user feedback, with the majority of ratings falling within the “Good” category. Specifically, the ease of application to the bleeding site was rated as “Good” in 85.7% (24/28) of cases, “Excellent” in 7.1% (2/28), and “Poor” in 7.1% (2/28). Conformance to the tissue surface was also rated “Good” by 85.7% (24/28), while 10.7% (3/28) rated it “Poor” and only 3.6% (1/28) found it “Excellent.” The ease of delivery to reach the wound site was considered “Good” in 96.4% (27/28) and “Excellent” in 3.6% (1/28) of the cases, with no reports of poor accessibility. Remarkably, the ease of preparation for use was uniformly rated as “Good” in 100% of cases, indicating a high degree of consistency and simplicity in product preparation. Although validated instruments were not used for reporting surgeon feedback, data were collected prospectively using standardized case record forms to maintain consistency in reporting. These findings suggest that the product was generally considered practical and user‐friendly by participating surgeons. Figure 3 presents the distribution of surgeon‐reported handling ratings for Surgi‐ORC across key intraoperative usability parameters.

## 4. Discussion

The present study provides preliminary observational data on the use of Surgi‐ORC in gynecological surgeries, demonstrating effective hemostasis and a short‐term safety profile. Hemostasis was achieved in all cases, with a mean TTH of 1.3 min, indicating rapid bleeding control across the study population. These findings are broadly consistent with a previous case report on Surgi‐ORC, where bleeding control is typically achieved within a few minutes of application without requiring additional interventions [15]. Moreover, studies evaluating other ORC materials such as Surgicel and Merizelle have also reported successful hemostasis within 2–3 min across gynecological surgical procedures [16, 17]. Although direct comparisons are not appropriate due to differences in study design and patient populations, the observed TTH in this study falls within the expected range reported in the literature.

Descriptive evaluation of TTH across product variants showed some variability; however, these differences should be interpreted cautiously due to the small and uneven subgroup analysis in the present study. In particular, the Fibril variant was used in only one case, making meaningful comparison with other variants unreliable. Therefore, no definitive conclusions were drawn regarding performance between product variants. Descriptive differences observed across product variants may be related to differences in material structure and surface characteristics. Previous studies have suggested that changes in surface area and charge density can influence hemostatic performance [18], while structural features such as fibrous arrangement may affect fluid absorption and interaction with clotting factors [19, 20]. These mechanisms, however, were not directly evaluated in the present study.

Descriptive evaluation across surgical procedures revealed that TTH values varied by specific gynecological procedures. Although, given the small and uneven subgroup sizes and limited representation of several gynecological procedure types, these results should be considered exploratory. Prior literature suggests that factors such as tissue vascularity, intraoperative blood loss, surgical technique, and patient‐specific characteristics play an important role in hemostatic outcomes [21, 22] and may support the variability observed in this study. These findings emphasize the need for procedure‐specific selection of the Surgi‐ORC variant, tailored for bleeding severity and anatomical site involvement.

From a safety perspective, no device‐related adverse events or clinically evident complications were observed during the 60‐day follow‐up period, indicating no short‐term safety concerns within the study population. Similarly, Shaltout et al. reported that Surgicel significantly reduces the recurrence of endometriomas; when used during laparoscopic drainage, it serves as an effective alternative to cystectomy with minimal impact on ovarian reserve [23]. Postoperative X‐ray imaging did not reveal abnormalities suggestive of retained material; however, plain radiography is not a validated method for confirming absorption of ORC. For this reason, the imaging findings should not be interpreted as confirmation of material absorption. Instead, the judgement was based on an X‐ray assessment by the operating surgeon and radiologist together with the established biodegradation profile of ORC [24].

Surgeon‐reported usability was generally favorable, with most handling parameters rated as “Good.” Similar observations have been reported with other topical hemostatic agents, where ease of use contributes to clinical adoption. However, in this study, usability was assessed using a nonvalidated, subjective questionnaire and should be interpreted accordingly.

While interpreting findings, several study limitations should be considered. In the TTH assessment, several procedure and device‐variant categories contained only one or two patients, precluding reliable subgroup comparisons or inferential modelling. The single‐arm design without a control group restricts the ability to draw comparative conclusions. Furthermore, the short‐term 60‐day follow‐up period provided may not be sufficient to assess long‐term outcomes such as adhesion formation.

Overall, this study provides preliminary insights on the effectiveness and safety of Surgi‐ORC used in gynecological surgeries. Larger, well‐designed studies with balanced cohorts, independent investigators, and comparative designs are needed to confirm these findings.

## 5. Conclusion

This prospective, multicentre, observational pilot study provides preliminary evidence that Surgi‐ORC was associated with achieving intraoperative hemostasis effectively, along with a short‐term safety profile in gynecological surgical procedures. Participating surgeons reported Surgi‐ORC as practical to use during evaluated procedures; however, these outcomes were based on subjective assessments using a nonvalidated questionnaire and should be interpreted accordingly. The safety findings should be inferred as the short‐term postoperative safety outcomes rather than long‐term complications such as adhesion formation, delayed foreign‐body reactions, or other late postoperative events. Given the exploratory nature of this study, the small sample size, uneven subgroup distributions and the absence of a comparator group, these findings should be interpreted with caution. Further well‐designed comparative studies with larger patient populations, independent investigators, longer follow‐up, and validated usability assessments are warranted to validate these results and explore broader clinical applications.

## Funding

This study was funded by Aegis Lifesciences Private Ltd., Gujarat, India, for overall research work. All participating institutions received funding from Aegis.

## Disclosure

Aegis Lifesciences Private. Ltd. was involved in study design, protocol development, coordination, and manuscript preparation. The authors were also involved in the study conception, data interpretation, and manuscript preparation. However, the clinical procedures, patient selection, intraoperative decision‐making, and outcome assessments were performed independently by the investigators at the participating centres in accordance with standard clinical practice. The sponsor had no role in influencing clinical decisions at the clinical site level. An independent third‐party clinical research organization (CRO) performed data collection, monitoring, verification, and statistical analysis to ensure objectivity and data integrity. The investigator has reviewed and approved the final manuscript.

Piyush Patel affirms that this manuscript is an honest, accurate, and transparent account of the study being reported; that no important aspects of the study have been omitted; and that any discrepancies from the study as planned (and, if relevant, registered) have been explained.

## Conflicts of Interest

All authors are full‐time employees of Aegis Lifesciences Private Ltd., Gujarat, India, the manufacturer of Surgi‐ORC, the product evaluated in this study.

## Supporting Information

Additional supporting information can be found online in the Supporting Information section.

## Supporting information


**Supporting Information** STROBE_checklist_v4_combined.

## Data Availability

The data that support the findings of this study are available on request from the corresponding author. The data are not publicly available due to privacy or ethical restrictions.
